# Concurrent changes in physical activity and physical functioning during retirement transition–a multi-trajectory analysis

**DOI:** 10.1371/journal.pone.0293506

**Published:** 2023-10-26

**Authors:** Roosa Lintuaho, Mikhail Saltychev, Jaana Pentti, Jussi Vahtera, Sari Stenholm

**Affiliations:** 1 Department of Emergency Medicine, Turku University Hospital and University of Turku, Turku, Finland; 2 Department of Physical and Rehabilitation Medicine, Turku University Hospital and University of Turku, Turku, Finland; 3 Department of Public Health, University of Turku and Turku University Hospital, Turku, Finland; 4 Centre for Population Health Research, University of Turku and Turku University Hospital, Turku, Finland; 5 Clinicum, Faculty of Medicine, University of Helsinki, Helsinki, Finland; 6 Research Services, Turku University Hospital and University of Turku, Turku, Finland; Clinca Geriatrica, ITALY

## Abstract

**Background:**

Physical activity and physical functioning have been reported to change over retirement transition, but the results have been inconsistent, and the two constructs have not been studied concurrently. The objective of this study was to examine concurrent changes in physical activity and physical functioning during transition to retirement among public sector employees, and to examine if occupation, sex, marital status, body mass index (BMI), alcohol consumption and smoking status are associated with observed different multi-trajectory paths.

**Methods:**

3,550 participants of the Finnish Retirement and Aging study responded to an annual survey on physical activity and physical functioning (SF-36) before and after retirement. Group-based multi-trajectory analysis was used to identify clusters with dissimilar concurrent changes in physical activity and physical functioning. Multinomial regression analysis was used to describe the associations between covariates and the probability of being classified to a certain cluster.

**Results:**

Participants were 63.4 (SD 1.4) years old, 83% women, 65% professional workers. Four trajectories of concurrent changes in physical activity and physical functioning were identified, one with decreasing physical functioning and low activity, one with increasing high activity and stable high functioning and two with fluctuating moderate physical activity and stable high functioning. Single, women, obese participants and risk-users of alcohol were more likely to be classified into group with low physical activity and declining physical functioning.

**Conclusions:**

Low physical activity below the level usually recommended was associated with lower physical functioning during retirement transition. These findings could be useful when planning interventions for retirees to maintain their physical functioning level.

## Introduction

Transition to retirement may change daily routines since work no longer dominates everyday schedule. There might be more leisure time for physical activity, hobbies and taking care of one’s health, although absence of daily routines provided by working schedules may have a passivating effect. Retirement has been reported to reduce headache [1] and low back pain [2] and improve sleep [3], which might improve physical functioning.

A systematic review on physical activity and retirement transition concluded that leisure-time physical activity increases after retirement [4]. A Finnish study has observed a temporary increase in self-reported leisure-time physical activity during retirement transition [5] with greater increase in physical activity among those with higher socioeconomic status and fewer chronic conditions. A French study has also reported increased self-reported leisure-time physical activity after retirement, especially among women [6]. Moreover, an accelerometer-based study from Finland has reported decrease in total daily activity after retirement among women who retired from manual work, while no change was observed among women who retired from non-manual work [7]. Men retiring from non-manual work increased their total daily physical activity, while activity remained the same for men retiring from manual work. Active commuters, e.g., cyclists and walkers, were more active both before and after retirement than other commuters and they also preserved their physical activity level after retirement [7].

There have been some studies regarding physical functioning and retirement, but the results have been inconsistent. In a Finnish study male managers have been followed around their retirement and five trajectories of physical functioning were identified using the 36-Item Short Form Health Survey (SF-36): “intact” (9%); “high and stable” (32%); “high and declining”(30%); “intermediate and declining”(24%) and “consistently low”(6%) [8]. Another Finnish study has followed women around their retirement transition reporting decrease in physical functioning associated with older age but without any visible effect of retirement [9]. In that study, only women who had a lower socioeconomic status and who retired due to illness showed improvement in physical functioning. A British study has observed decline in physical functioning with aging [10], whereas a Finnish study has reported decrease in physical functioning among those retired due to illness, while the level of physical functioning has increased or remained the same for the other participants [11]. In that study, higher occupational position and lower physical activity were associated with greater decrease in physical functioning after retirement.

While both physical activity and physical functioning have been reported to change over retirement transition, observations have been inconsistent [4–6, 9–13], and the reports on their concurrent changes have been missing. Association between changes in physical activity and functioning could, however, be expected as better functioning may enable being physically active as well as physical activity may help to maintain or improve physical functioning. Indeed, there is some evidence that physical activity could be protective of functional limitations and chronic pain [14, 15] and the associations between physical activity and cardiovascular disease have clearly been established [16].

The objective of this study was to examine concurrent changes in physical activity and physical functioning during transition to retirement among public sector employees. Multi-trajectory analysis was used to examine whether there are subgroups with different trajectory paths during this transition. The secondary aim was also to examine whether occupation, sex, marital status, body mass index (BMI), alcohol consumption or smoking status predict the probability of being classified to a certain subgroup, since these factors have been linked to both physical activity and physical functioning [13, 14, 17, 18].

## Methods

### Study cohort

The Finnish Retirement and Aging Study (FIREA) is an ongoing longitudinal cohort study, which follows aging workers from final years in working life to full-time retirement and until old age [19]. The eligible population for the FIREA included all public sector employees whose individual retirement date was between 2014 and 2019 and who were working in the year 2012 in one of 27 municipalities, nine cities or five hospital districts. Information on the estimated individual retirement date was obtained from the register kept by the pension provider for public sector employees (Keva).

**Fig 1:**
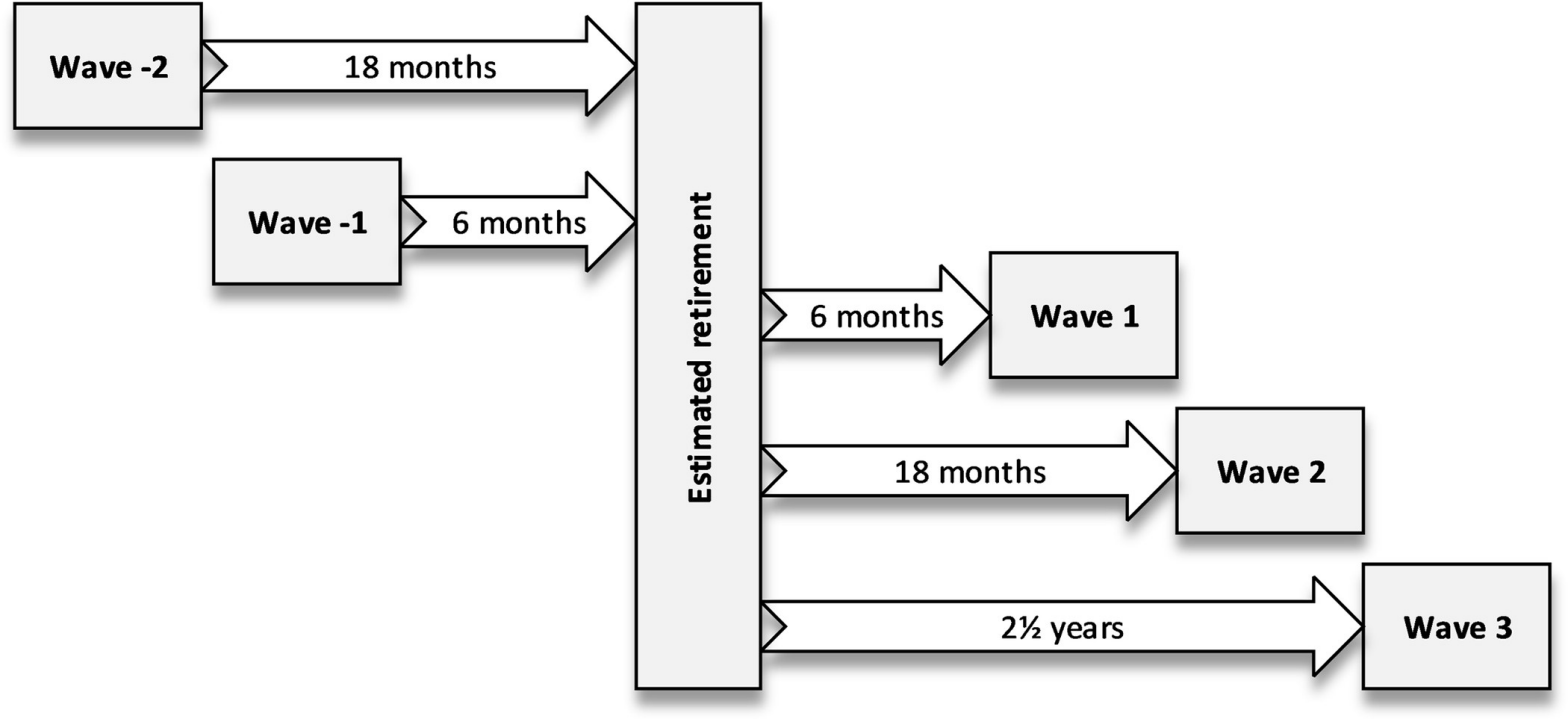
Study waves and follow-up time.

The participants were first contacted 18 months prior to their estimated retirement date by a questionnaire, which has thereafter been sent annually, at least four times. The actual retirement date was reported by the participants. Participants who have provided information regarding physical activity and physical functioning at least two consecutive times, one right before retirement and one after, were included in the current study (n = 3,550). There were two possible survey waves before the retirement, waves -2 and -1, and three possible waves after the retirement, waves 1, 2 and 3 (Fig 1).

All the respondents have provided a written informed consent. The FIREA is following the Declaration of Helsinki and has been approved by the Ethics Committee of Hospital District of Southwest Finland.

### Measurement of physical activity and physical functioning

Physical activity was self-reported at each study wave. The participants were asked to estimate their average weekly hours of leisure-time physical activity (including commuting) [20]. The level of physical activity was then converted into metabolic equivalent of task (MET), which describes the amount of consumed energy comparing to resting. One MET unit of 3.5 ml/kg/minute corresponds to oxygen consumption while sitting at rest. Weekly physical activity was expressed as MET-h/week. For the interpretation of the results following categorization was used: low (<14 MET-h/week), moderate (14 to <30 MET-h/week) or high (≥30 MET-h/week) physical activity levels [20, 21]. This categorization was chosen since physical activity lower than 14 MET-h/week has been reported to be associated with higher risk of cardiovascular disease [22] and the activity level of 30 MET-h/week has been shown to be needed for succesful weight management [23]. 14 MET-h/week are approximately the equivalent to 140 minutes and 30 MET-h/week to 300 minutes of brisk walking weekly.

Physical functioning was assessed using the SF-36 survey [24], which is a 36-item set of generic quality of life measures that has been translated to Finnish [25]. Of the survey’s 36 questions 10 concern physical functioning as a subscale, and those were included in the study. The composite score was calculated as a sum of averages of individual answers. Thus, a composite score ranged from 0 to 100 points with higher score describing better physical functioning. The assessment of physical activity and physical functioning is presented in S1 Table.

### Covariates

Age was defined in years. Marital status was dichotomized as “married or co-habiting” vs. “single”. Occupational titles were obtained from the register of pension provider (Keva), and they were coded according to the International Standard Classification of Occupations (ISCO-08) and dichotomized as “professionals” (ISCO major groups 1–4) vs. “service and manual workers” (ISCO major groups 5–9). Body mass index (BMI) was calculated as height/weight^2^ according to self-reported body height and weight. BMI was dichotomized as “normal and overweight” (BMI < 30kg/m^2^) vs. “obesity” (≥30kg/m^2^). Smoking was dichotomized as “current smokers” vs. “never-smokers and former smokers”. Alcohol consumption was obtained from the survey as units of alcohol consumed weekly and converted into grams of pure alcohol per week (g/week). Amount of >288 g/week for men and >192 g/week for women was considered a cut-off for excess alcohol consumption and dichotomized as “risk-users “vs. “non-risk-users”. Information on covariates were obtained at the last available wave before retirement, wave -1.

### Statistical analysis

The descriptive statistics were reported as means and standard deviations or as absolute numbers and percentage, when appropriate.

The group-based multi-trajectory analysis (GBTA) was used to investigate the developmental trajectories (a course of outcome over time) of physical activity and physical functioning. The group-based multi-trajectory analysis is a form of finite mixture modelling for analysing longitudinal repeated measures data [26, 27]. The GBTA is a simpler subclass of the growth mixture modelling (GMM). The difference between two approaches is that the GMM allows for within-class variation, while the GBTA assumes that all individuals in a trajectory class have the same behavior. While the GBTA estimates fewer parameters, it runs faster, with fewer errors and the results may be easier to interpret compared to more advanced GMM. It has been suggested that the GBTA may usually be the more practical choice [28]. While conventional statistics show a trajectory of average change of outcome over time, the group-based trajectory modelling is able to distinguish and describe subpopulations (clusters) existing within a studied population. Multi-trajectory modelling defines the trajectories of multiple outcomes simultaneously [27]. The trajectories of such subpopulations may differ substantially from each other and from the average trajectory observed in the entire population. The censored (known also as ‘regular’) normal model of the group-based multi-trajectory analysis was used. The goodness of model fit was judged by running the procedure several times with a number of clusters starting from one up to five. The Bayesian Information Criterion (BIC), Akaike information criterion (AIC) and average posterior probability (APP) were used as criteria to confirm the goodness of fit. Linear, quadratic and cubic regression models were tested and cubic model was retained for using in the analysis. The cut-off for the smallest group was set at ≥5% of the entire cohort. Physical activity (MET-h/week) and physical functioning (SF-36 score) were simultaneously used as two separate variables in their continuous form. The changes in both variables were presented graphically by each of identified trajectory groups (clusters).

A multinomial regression analysis was used to describe the associations between demographic and lifestyle factors and probability of being classified into a particular cluster. The results were presented as relative risk ratios (RRRs) and their 95% confidence intervals (95% CIs). The RRRs were adjusted for age and sex.

The analyses were performed using Stata/IC Statistical Software: Release 16. College Station (StataCorp LP, TX, USA). The additional Stata module ‘traj’ was required to conduct group-based trajectory analysis. The module is freely available for both SAS® and Stata software [29].

## Results

The pre-retirement descriptive characteristics of the study population are presented in Table 1. Of the respondents, 83% were women and mean age at the last wave before retirement was 63.4 years (SD 1.4). Before the retirement, 65% were professionals, 69% were married or co-habiting, 28% were obese, 9% current smokers and 2% alcohol risk users.

**Table 1:** Pre-retirement characteristics of the study population (wave -1).

	n	%
**Sex**		
Women	2949	83
Men	601	17
**Marital status**		
Single	1088	31
Co-habiting	2462	69
**ISCO class**		
Professional	2283	65
Manual or service worker	1239	35
**BMI**		
BMI <30	2569	72
BMI >30	981	28
**Smoking**		
Not currently	3192	91
Currently smoking	305	9
**Alcohol consumption**		
No risk-use	3457	98
Risk-use	87	2
**Age**	**Mean**	**SD**
	63.4	1.4

### Trajectory groups

**Fig 2:**
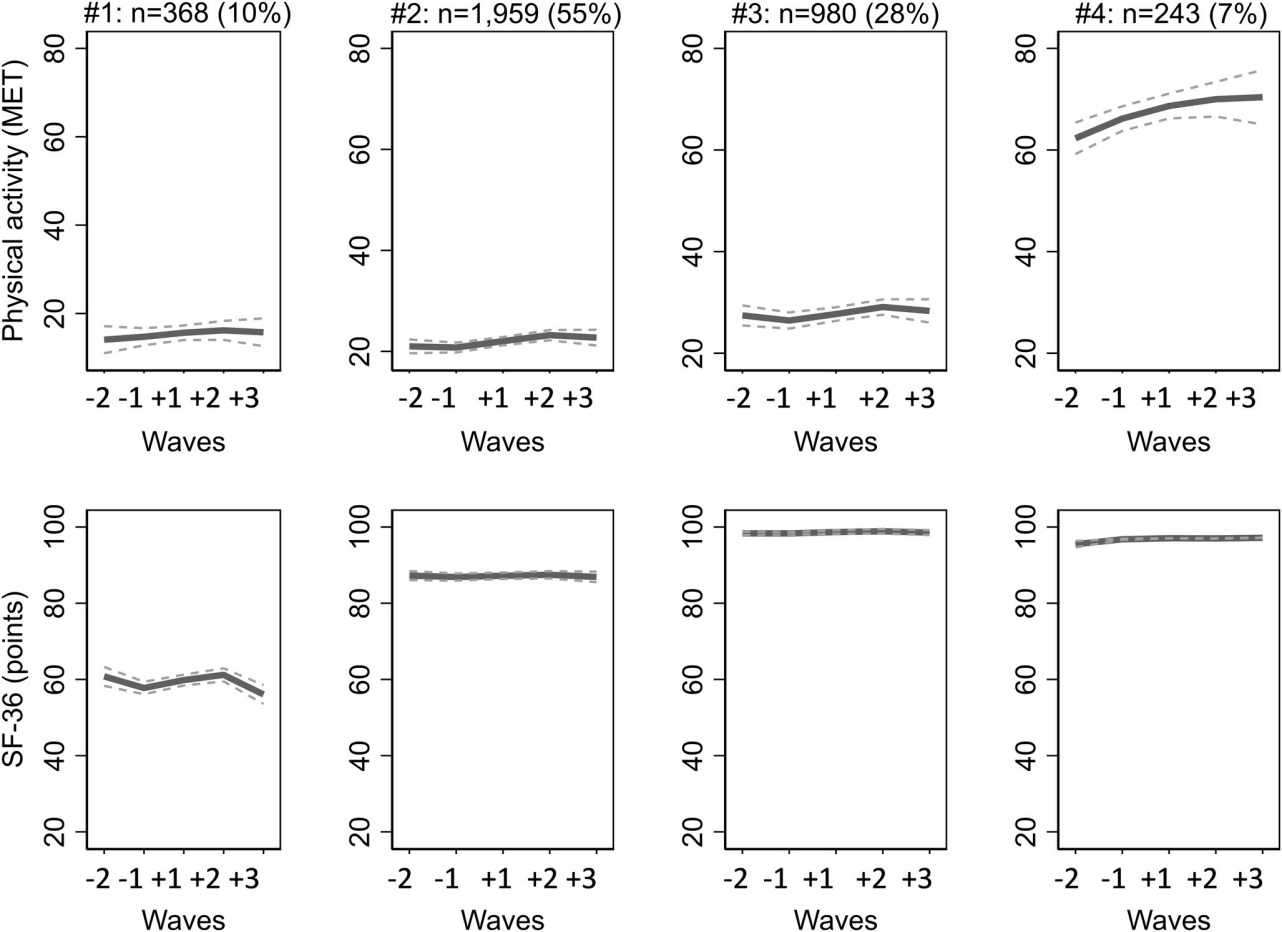
Concurrent trajectories of physical activity and physical functioning in five study waves during retirement transition.

A four-trajectory model was chosen as the smallest group in the five-trajectory model fell below a pre-agreed cut-off of 5% (Table 2). The smallest average posterior probability (APP) for a four-trajectory model was sufficient 0.89. Also, Bayesian Information Criterion (BIC) and Akaike information criterion (BIC) were closer to zero for a four-trajectory model than for the models with smaller number of trajectory groups. Following four concurrent trajectory groups of physical activity and physical functioning were identified (Fig 2).

**Table 2:** Goodness of fit of group-based trajectory analysis models. The chosen model is shown in bold.

Number of clusters	Shape of trajectory	Smallest group	BIC^1^	AIC^2^	APP^3^
1	Cubic	3,550	103,735	103,694	1.0
2	Cubic	952	101,470	101,392	0.92
3	Cubic	310	99,682	99,567	0.93
**4**	Cubic	**239**	**98,844**	**98,667**	**0.89**
5	Cubic	110	98,532	98,311	0.88

^1^ BIC = Bayesian Information Criterion

^2^ AIC = Akaike information criterion

^3^ APP = Smallest average posterior probability

### Group #1: Low physical activity and declining physical functioning (10%)

Physical activity was constantly low. Before retirement physical activity was 12 MET-h/week, then increased during retirement transition up to 14.9 MET-h/week, but eventually decreased to a level of 14.2 MET-h/week after retirement. Physical functioning was at a moderate level of 59.9 points before retirement and then mildly increased during retirement transition up to 60.7 points and decreased after the retirement to 56.1 points.

### Group #2: Moderate physical activity and steady high physical functioning (55%)

Moderate physical activity of 20 MET-h/week before retirement slightly increased during retirement transition to 22.4 MET-h/week. Physical functioning remained steadily at a high level from 87.3 to 87.9 points before and after retirement.

### Group #3: Moderate physical activity and steady excellent physical functioning (28%)

Physical activity was on moderate level of 27.7 MET-h/week two years before retirement, then decreased before retirement to 25.7 MET-h/week and increased during retirement transition to 28.5 MET-h/week after retirement. In turn, physical functioning remained steady, being 98.6 to 99.1 points during the follow-up.

### Group #4: Increasing high physical activity and steady excellent physical functioning (7%)

High physical activity of 63.2 MET-h/week before retirement further increased during retirement transition to 71.3 points and after retirement remained at 68.3 to 70.7 points. Physical functioning remained at almost maximal level, ranging from 94.9 to 97.0 points, throughout the retirement transition years.

The average estimates of physical activity and physical functioning at different study waves are shown by trajectory groups in S2 Table.

### Associations between covariates and trajectory groups

To compare characteristics of the trajectory groups, group #2 (moderate physical activity and steady high physical functioning) was used as a reference group (Table 3). Compared to group #2, there were more women in group #1 (RRR 1.55, 95%CI 1.09–2.20) and more men in groups #3 (RRR 0.80, 95%CI 0.66–0.98) and #4 (RRR 0.57, 95%CI 0.42–0.79). There were fewer married or co-habiting participants in group #1 (RRR 0.76, 95%CI 0.60–0.96) and more in group #3 (RRR 1.27, 95%CI 1.07–1.51) than in group #2. Manual or service workers were less likely to be classified to groups #3 (RRR 0.77, 95%CI 0.66–0.91) or #4 (RRR 0.72, 95%CI 0.53–0.97) than in group #2. There was no difference in occupational status between groups #1 and #2. Obese participants (RRR 3.27, 95%CI 2.60–4.12) and smokers (RRR 1.67, 95%CI 1.20–2.33) were more likely to be categorized into group #1 than group #2. On the other hand, obesity was less common in group #3 (RRR 0.32, 95%CI 0.26–0.40) and #4 (RRR 0.24, 95%CI 0.15–0.37), and smoking in group #4 (RRR 0.44, 95%CI 0.23–0.84) than group #2. Alcohol risk use was more common in group #1 (RRR 2.24, 95% CI 1.30–3.86) than in group #2, and participants who were risk-users were less likely to be in group #3 (RRR 0.42, 95% CI 0.22–0.79) than in group #2.

**Table 3:** Relative risk ratios (RRRs) of being classified to a certain trajectory group, group #2 used as a reference.

**Variable/group**	**RRR**	**95% CI**	
**Women vs. men**			
Group #1: Low physical activity and declining physical functioning	1.55	1.09	2.20
Group #2: Moderate physical activity and steady high physical functioning	1.00		
Group #3: Moderate physical activity and steady excellent physical functioning	0.80	0.66	0.98
Group #4: Increasing high physical activity and steady excellent physical functioning	0.57	0.42	0.79
**Married or co-habiting vs. single**			
Group #1: Low physical activity and declining physical functioning	0.76	0.60	0.96
Group #2: Moderate physical activity and steady high physical functioning	1.00		
Group #3: Moderate physical activity and steady excellent physical functioning	1.27	1.07	1.51
Group #4: Increasing high physical activity and steady excellent physical functioning	1.29	0.95	1.74
**Manual or service workers vs. professional workers**			
Group #1: Low physical activity and declining physical functioning	1.14	0.91	1.43
Group #2: Moderate physical activity and steady high physical functioning	1.00		
Group #3: Moderate physical activity and steady excellent physical functioning	0.77	0.66	0.91
Group #4: Increasing high physical activity and steady excellent physical functioning	0.72	0.53	0.97
**BMI ≥30 vs. BMI <30 kg/m** ^**2**^			
Group #1: Low physical activity and declining physical functioning	3.27	2.60	4.12
Group #2: Moderate physical activity and steady high physical functioning	1.00		
Group #3: Moderate physical activity and steady excellent physical functioning	0.32	0.26	0.40
Group #4: Increasing high physical activity and steady excellent physical functioning	0.24	0.15	0.37
**Current smokers vs. former or never smokers**			
Group #1: Low physical activity and declining physical functioning	1.67	1.20	2.33
Group #2: Moderate physical activity and steady high physical functioning	1.00		
Group #3: Moderate physical activity and steady excellent physical functioning	0.75	0.56	1.01
Group #4: Increasing high physical activity and steady excellent physical functioning	0.44	0.23	0.84
**Risk-users of alcohol vs. non-risk users**			
Group #1: Low physical activity and declining physical functioning	2.24	1.30	3.86
Group #2: Moderate physical activity and steady high physical functioning	1.00		
Group #3: Moderate physical activity and steady excellent physical functioning	0.42	0.22	0.79
Group #4: Increasing high physical activity and steady excellent physical functioning	0.38	0.12	1.23

RRRs are adjusted for age and sex (only age for sex RRR).

## Discussion

This prospective cohort study amongst 3,550 public sector employees investigated concurrent changes in physical activity and physical functioning during retirement transition by using a group-based multi-trajectory analysis. Four trajectory groups were identified, displaying different levels and patterns of physical activity and physical functioning before and after retirement.

Low activity below the current activity recommendation (14 MET-h/week) [30, 31] was clearly associated with poorer physical functioning, since all the trajectory groups in which physical activity was above 14 MET-h/week showed better physical functioning. Interestingly, there was no substantial difference between groups in the level of physical functioning once the level of physical activity has exceeded the level of approximately 20 MET-h/week–the score for physical functioning varied between 90 and 98 points in all three groups with moderate (20 MET-h/week) or high (30 MET-h/week to 60 MET-h/week) physical activity.

In line with some previous studies, this study demonstrated an increase in physical activity during retirement transition, although the changes in most groups were small [4–6]. Also, as shown previously, the identified trajectories displayed minor increase in physical activity around the time of retirement, but stable or even slightly declining physical activity after that [5]. Only group #4 demonstrated different trajectory patterns showing initially very high physical activity, which increased throughout the follow-up. It should be noticed that this group was relatively small, including only 7% of the participants and they were exceptionally physically active–indeed, 60 MET-h/week is an amount approximately equivalent to 10 weekly hours of brisk walking.

On contrary to the present results, some previous studies have observed decline in physical functioning after retirement [10, 13], and some have found rather stable or even improving physical functioning among older retirees [11, 12]. In the current study, physical functioning was declining prior to and after the retirement among participants with initially poorer physical functioning, but participants with initially high functioning preserved their high level throughout the follow-up. In groups #3 and #4 the level of physical functioning was already very high measured by the SF-36, that no improvement was possible using the current meter. In group #2, improvement of functioning could have been possible, but even though the participants were active, they did not improve their functioning level. Thus the hypothesis that increasing physical activity could further increase physical functioning was not supported by the current study. The changes in physical functioning were small and inconsistent. A possible reason for that is that initially most of the participants were in good health without any substantial limitations of physical functioning, and the rough SF-36 can only identify larger restrictions in physical functioning, thus not much change was observed.

There were also some differences between the identified groups regarding sociodemographic and lifestyle factors. Group #2 (moderate physical activity and steady high physical functioning) was used as a reference group, because it was the largest group and it included participants with high level of functioning who meet current recommendations for physical activity. Single, women, obese participants, smokers and risk-users of alcohol were more likely to be classified into group with low physical activity and declining physical functioning compared to the reference group #2. In turn, married or co-habiting, men, professional workers, non-risk-users of alcohol and normal or overweight participants were more likely included into groups with moderate or increasing physical activity and steady excellent physical functioning compared to reference group #2. These observations are in line with previous research. Male sex has been found to be associated with better functioning [13], although associations between sex and physical activity have been inconclusive [6, 7]. When it comes to occupation, retiring from manual work has been associated with declining total physical activity and retiring from sedentary work with increasing total physical activity [7, 32]. Higher socioeconomic status has been constantly associated with higher physical activity [33–35] and physical functioning [13], although there has been some evidence that the decline in functioning after retirement might be greater for those with higher occupational grade [11]. Finally, higher BMI and obesity have been reported to be associated with lower physical activity and greater risk of physical disability [36].

### Strengths and limitations

The strengths of this study include annually repeated measurements of physical activity and physical functioning before and after the transition to retirement in a large sample of public sector employees covering a wide range of occupations. Physical activity and physical functioning as well as BMI, were self-reported, which might lead to information bias [37]. The SF-36 survey provides only a rough measure of physical functioning. It is possible to get almost full points from the survey and still have restrictions that affect the ability to move and exercise, and persons who retire due to old age can still be in so good shape, that the meter can’t distinguish differences. A more detailed meter could have been useful to detect the small changes in physical functioning.

Due to the study setting, the participants were probably healthier and better functioning than a general population as they were mostly those who are retiring due to an old age, leaving out persons who are retiring due to poor health condition. Participants who retired due to illness were not specified in the study, but since all the participants were still working 18 months prior to their planned retirement date, the number of these retirees was small. The participants were mostly permanently employed. Reflecting a common sex distribution in a public sector (many female-dominated professions, such as nurses or teachers), the studied cohort was predominated by women. Also, 65% of the cohort were professional workers. These factors limit the generalisability of the results on general population. The study only included commuting and leisure-time physical activity, leaving out work-related activity. Some occupations are more physically demanding than others, yet these differences were not detailed in the study. Retiring from physically hard work could relieve musculoskeletal health problems, such as back or knee pains, which might affect physical functioning as well as physical activity. Only the initial estimates were used for the predictive covariates in the analysis. Thus, potential fluctuation in the covariates during the follow-up were not taken into account.

Further research is needed to understand better how physical demands of work affect physical activity and functioning after retirement and how to activate people during retirement transition years so that they could preserve their physical functioning.

## Conclusions

Four groups with different patterns of physical activity and physical functioning were identified. Physical activity below the level usually recommended was associated with lower physical functioning and those with high physical activity showed consistently high physical functioning. Single, women, obese participants, risk-users of alcohol and smokers were more likely to be classified into group with low physical activity and declining physical functioning. These findings could be useful when planning and targeting health interventions for people in their retirement age.

## Supporting information

S1 Table The assessment of physical activity and physical functioning. (PDF)

S2 Table The average estimates of physical activity and physical functioning at different study waves by trajectory groups. (PDF)
