# Supplementary material for: Concurrent changes in physical activity and physical functioning during retirement transition–a multi-trajectory analysis
Source: PLoS One. 2023 Oct 26;18(10):e0293506. doi: 10.1371/journal.pone.0293506 (PMC10602340; doi:10.1371/journal.pone.0293506)
Supplement: S1 Table — (PDF) [file pone.0293506.s001.pdf]

Supplement 1. Assessment of physical activity and physical functioning

Physical activity				
Average weekly hours spent among activites correspongind to				
	Not at all	<30 minutes	2 to 3 hours	> 4 hours
Walking				
Brisk walking				
Jogging				
Runnin				
Physical functioning				
How much limitation there is caused by health problems in following tasks				
		A lot	A litte	Not at all
Vigorous activities, i.e. running, heavy lifting				
Moderate activites, i.e. vacuum cleaning				
Carrying or lifting groceries				
Climbing several flight of stairs				
Climbing one flight of stairs				
Bending, kneeling or stooping				
Walking more than a mile				
Walking several blocks				
Walking one block				
Bathing or dressing				
