# Supplementary material for: Concurrent changes in physical activity and physical functioning during retirement transition–a multi-trajectory analysis
Source: PLoS One. 2023 Oct 26;18(10):e0293506. doi: 10.1371/journal.pone.0293506 (PMC10602340; doi:10.1371/journal.pone.0293506)
Supplement: S2 Table — (PDF) [file pone.0293506.s002.pdf]

Supplement 2. The level of physical activity and physical functioning at different waves by trajectory groups

Study waves	Group #1: Low physical activity and declining physical functioning (10%)			Group #2: Moderate physical activity and steady high physical functioning (55%)			Group #3: Moderate physical activity and steady excellent physical functioning (28%)			Group #4: Increasing high physical activity and steady excellent physical functioning (7%)		
	Mean	95% CI		Mean	95% CI		Mean	95% CI		Mean	95% CI	
Physical activity, MET/week												
-2	12.0	10.4	13.7	20.1	19.2	20.9	27.7	26.4	29.0	63.2	58.5	67.8
-1	12.8	11.7	13.9	19.6	19.0	20.2	25.7	24.7	26.7	65.8	61.8	69.7
1	13.9	12.6	15.3	21.4	20.8	22.0	28.4	27.4	29.3	71.3	67.3	75.3
2	14.9	13.5	16.3	22.4	21.7	23.1	28.3	27.2	29.3	68.3	63.3	73.3
3	14.2	12.4	16.0	22.2	21.2	23.2	28.5	26.9	30.1	70.7	62.8	78.5
Physical functioning, SF-36 points												
-2	59.9	56.8	63.0	87.8	87.2	88.4	98.7	98.5	98.9	94.9	93.3	96.5
-1	55.9	53.9	57.9	87.3	86.9	87.8	98.6	98.4	98.8	96.4	95.5	97.2
1	60.7	58.8	62.7	87.9	87.5	88.4	99.1	99.0	99.3	97.0	96.3	97.8
2	59.6	57.2	62.0	87.9	87.4	88.5	99.1	98.9	99.3	97.0	96.2	97.8
3	56.1	53.1	59.1	87.7	86.9	88.4	98.8	98.6	99.1	97.0	95.7	98.3
